# Preferred intensity exercise for adolescents receiving treatment for depression: a pragmatic randomised controlled trial

**DOI:** 10.1186/s12888-015-0638-z

**Published:** 2015-10-14

**Authors:** Tim Carter, Boliang Guo, David Turner, Ioannis Morres, Elizabeth Khalil, Emily Brighton, Marie Armstrong, Patrick Callaghan

**Affiliations:** School of Health Sciences, University of Nottingham, Nottingham, UK; Division of Psychiatry & Applied Psychology, University of Nottingham, Nottingham, UK; School of Medicine, University of East Anglia, East Anglia, UK; Department of Physical Education & Sport Science, University of Thessaly, Trikala, Greece; Specialist CAMHS, Nottinghamshire Healthcare NHS Trust, Nottingham, UK

**Keywords:** Depression, Adolescence, Young people, Exercise, Physical activity, RCT

## Abstract

**Background:**

Exercise has been shown to be effective in treating depression, but trials testing the effect of exercise for depressed adolescents utilising mental health services are rare. The aim of this study was to determine the effectiveness of a preferred intensity exercise intervention on the depressive symptoms of adolescents with depression.

**Methods:**

We randomly assigned 87 adolescents who were receiving treatment for depression to either 12 sessions of aerobic exercise at preferred intensity alongside treatment as usual or treatment as usual only. The primary outcome was depressive symptom change using the Children’s Depression Inventory 2^nd^ Version (CDI-2) at post intervention. Secondary outcomes were health-related quality of life and physical activity rates. Outcomes were taken at baseline, post intervention and at six month follow up.

**Results:**

CDI-2 score reduction did not differ significantly between groups at post-intervention (est. 95 % CI −6.82, 1.68, *p* = 0.23). However, there was a difference in CDI-2 score reduction at six month follow-up in favour of the intervention of −4.81 (est. 95 % CI −9.49, −0.12, *p =* 0.03). Health-related quality of life and physical activity rates did not differ significantly between groups at post-intervention and follow-up.

**Conclusions:**

There was no additional effect of preferred intensity exercise alongside treatment as usual on depressive reduction immediately post intervention. However, effects were observed at six months post-intervention, suggesting a delayed response. However, further trials, with larger samples are required to determine the validity of this finding.

**Trial registration:**

ClinicalTrials.gov NCT01474837, March 16 2011

## Background

The prevalence of depression in adolescents has doubled between the mid-1980’s and 2000’s [1] with major Depressive Disorder (MDD) now ranging from four to eight %. Additionally, 12 % of children and adolescents may have sub threshold symptoms of depression [2] and 20 % of young people experience at least one episode of major depression before they reach 18 years of age [3].

In adults, exercise has been shown to improve mood, self-esteem, self-worth, anxiety and depression [4–8]. A recent Cochrane review suggests that exercise is likely to decrease depressive symptoms by the end of treatment and at long-term follow up [9]. However, when only methodologically robust trials were included in the analysis a smaller effect in favour of exercise was observed.

Numerous theories attempt to explain the exercise/depression relationship ranging from psychological theories such as the distraction hypothesis, the self-efficacy hypothesis and the mastery hypothesis [10] to more biochemical based theories such as the monoamine hypothesis, the endorphin response and the transient hypofrontality hypothesis [11]. However there appear to be no consensus as to the actual mechanism of action.

The effectiveness of exercise for reducing depression in adolescents has received substantially less attention than in adults. In a recent systematic review investigating the impact of physical activity on depression scores in children and adolescents, a small, statistically significant treatment effect was observed for the physical activity intervention group over the non-physical comparison [12]. However, the majority of reviewed trials recruited non-clinical populations. The findings of a similar systematic review of clinical trials up to 2005 [13] reported only three trials investigating the antidepressant effect of exercise on a clinical population. All three studies reported no significant difference in depression scores between the intervention and control groups. However, two of the studies were reported to be of low and moderate methodological quality, with all three studies having relatively small sample sizes.

Such findings may be attributed to the method of implementation, as the majority of exercise trials with adults and adolescents have typically implemented prescribed (fixed) intensity exercise interventions. However, observational studies in both adults and young people report that prescribed (fixed) intensity exercise results in less favourable affective responses and effort perceptions in comparison to preferred intensity exercise [14, 15]. Such findings have been replicated in a trial comparing preferred intensity exercise against prescribed intensity exercise for depressed adults [16]. Here, the authors reported increased antidepressant effects, lower effort perception and decreased attrition rates for those in the preferred intensity condition [16, 17].

In summary, there appears to be some, albeit limited, evidence that exercise may improve depression scores in adolescents. Additionally, there is evidence to suggest that preferred (non-prescribed) intensity exercise may be an effective method of exercise delivery. However, to the authors' best knowledge there has been no well-designed RCT's with large enough samples to determine the effectiveness of exercise for adolescents receiving treatment for depression.

### Aims

To determine the effectiveness of a preferred intensity exercise intervention on the depressive symptoms of adolescents with depression.

## Methods

### Study design

**Fig. 1:**
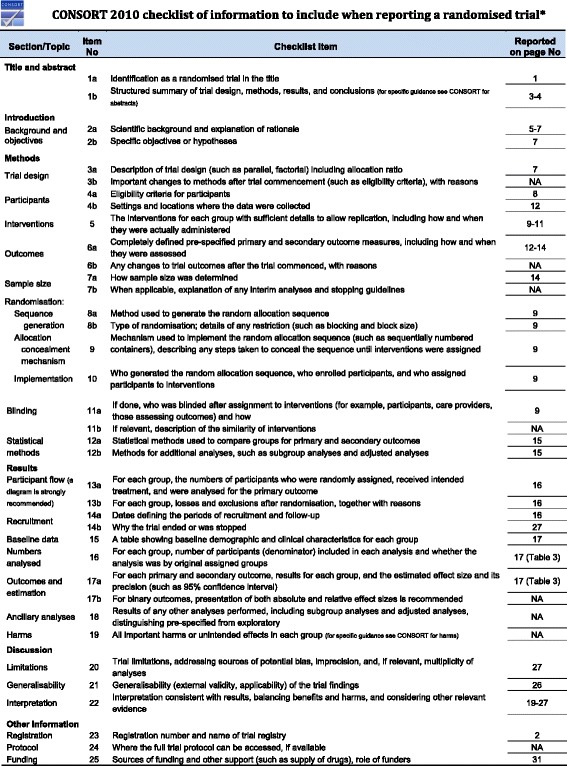
CONSORT checklist of information to include when reporting a randomised tria.l *We strongly recommend reading this statement in conjunction with the CONSORT 2010 Explanation and Elaboration for important clarifications on all the items. If relevant, we also recommend reading CONSORT extensions for cluster randomised trials, non-inferiority and equivalence trials, non-pharmacological treatments, herbal interventions, and pragmatic trials. Additional extensions are forthcoming: for those and for up to date references relevant to this checklist, see www.consort-statement.org

A pragmatic Randomised Controlled Trial (RCT) (parallel design) was used to compare the effectiveness of exercise on depression alongside treatment as usual (TAU) at post intervention (six weeks) and at six month follow up. Participants were individually randomised to an intervention arm in which they undertook a six week exercise intervention alongside TAU or to TAU only control arm. The extended CONSORT statement for the reporting of pragmatic trials [18] was adhered to in the reporting of this trial. For the completed CONSORT checklist please see Fig. 1.

### Recruitment

Participants were referred by General Practitioners (G.Ps), Child and Adolescent Mental Health Services (CAMHS) and school nurses within the East Midlands area of England.

Informed consent was obtained in writing from the legal guardians of young people under 16 years of age, alongside written assent from the young person. For young people aged 16 years and over, written informed consent was obtained from the young person themselves.

Participants were informed that all travel expenses to attend each exercise session would be reimbursed. Participants were also informed that they would be given a £15 high street voucher following the six-month data collection, when their participation in the study ended.

### Eligibility criteria

Inclusion criteria were adolescents aged 14–17 years, receiving treatment from a health or social care professional for depression, and scoring above 14 on the Children’s Depression Inventory-2 (CDI-2) (a recommended cut off point indicating clinical levels of depression) [19]. In order to make the RCT as generalizable as possible to routine clinical practice, the only exclusion criterion was the presence of a medical condition that would make exercise participation unsafe. This was assessed by the referring clinician and confirmed by the trial exercise therapist. As such, adolescents with self-harm injuries (e.g. treated with bandages) or various physical health problems including sciatica, chronic low back pain, knee joint injuries or severe hemicranias participated in the trial.

### Randomisation and allocation concealment

The random allocation sequence was computer generated by the trial statistician using permuted block randomisation with varying block size. In order to ensure allocation concealment sequentially numbered opaque sealed envelopes were used. Individuals were randomised to groups by a researcher not connected to the study team.

### Blinding

Outcome assessors, including the data input administrator, were blind to treatment group at both follow up time points. The analysis was conducted on a data set in which group allocation was unlabelled.

### The intervention

The intervention was a six week circuit-training consisting of 12 separate sessions which were run twice weekly. The circuit training comprised of an interval pattern with eight separate exercise-stations. The stations consisted of strengthening and aerobic exercises: abdominal and back exercises from the supine and prone positions respectively; two medicine ball arm-based exercises from supine position; bouncing, static and dynamic balance exercises on a trampoline; body-weight squat exercise against the wall and stationary cycling.

Following five minutes of stretching on major muscle groups in the upper and lower limbs, participants were encouraged to exercise for one minute then break for one minute, this was then repeated twice more. Subsequently, participants exercised for two minutes followed by a break of one minute; this was then repeated nine times. Subsequently, a five minute stretching exercise on major muscle groups closed the intervention.

The total duration of each session was one hour (approximately 45 min of exercise and stretching). A qualified exercise therapist supervised each session (IM). Two additional staff members of the project exercised and interacted with participants in all sessions including the first author (TC). Preferred intensity was operationalised as follows: Participants could choose the order in which they undertook the different exercises; they could choose the intensity in which they exercised on each station and they could choose to take rests when they wanted. Moreover, no pressure was applied to participants to exercise at higher levels nor was it applied to participants to attend the sessions.

Session attendance, self-perceived physical exertion and heart rate were used as measures of intervention adherence and engagement. The number of sessions each participant attended was recorded at each session using a paper register. We minimised the likelihood of participants witnessing the register being taken to reduce the potential for this to affect attendance.

Self-perceived physical exertion was measured using the Borg Rating of Perceived Exertion (RPE) Scale. The Borg RPE scale is a tool for estimating effort and exertion, breathlessness and fatigue during physical exercise [20]. Using the RPE scale respondents state how hard they are exercising on a scale ranging from 6 to 20 (‘no feeling of exertion’ to ‘very, very hard’ respectively). It is reported to be readily learned by adolescents and is a useful frame of reference for self-regulating exercise intensity [21]. While exercising, participants were asked to point out their exertion levels on the RPE scale at three time points in each exercise session.

Participants’ heart rate was monitored at five time points throughout each exercise session by means of heart rate wrist monitors to record physiological responses and to ensure exercise was taking place safely (≤80 % of the maximum heart rate -[220-age]). To measure heart rate, participants placed two fingers on separate panels of the faceplate and held for 5 s. The exercise therapist also visually verified heart rate from the watches. Each participant was given a heart rate monitor watch at the beginning of each session and asked to monitor and verbalise their heart rate to the exercise therapist at the specified time points. The exercise therapist also ascertained heart rate from the watches at each data collection point.

### TAU

The term TAU typically refers to a wide range of standard treatments available to the specific group in question. In this instance, TAU included psychological therapies and, in rare cases, pharmacotherapy (see Table 2) with no participants reporting exercise as part of their usual treatment.

### Outcome measures

Outcome measures were taken face-to-face at three time points: baseline (prior to randomisation), post intervention (six weeks) and at six month follow up. A modified Client Service Receipt Inventory (CSRI) was also collected at this time point to record resource use. Outcome measures were taken at a place designated by the participant (this was typically at the participants own home).

### Primary outcome measure

#### The Children’s depression inventory 2(CDI-2) [19]

The CDI-2 is a 28-item self-report questionnaire designed to assess the severity of current/recent depressive symptoms in adolescents aged 7 to 17. The CDI-2 includes items that cover the key criterion symptoms of depression with age appropriate manifestations of several symptoms in terms of both functional and affective features over the preceding two weeks [19]. The response options for each item are rated on a 3-point scale as follows: 0 (no symptom), 1 (probable or mild symptom), and 2 (definite, marked symptom). The range of scores is 0–56 with higher scores representing increased depressive symptom severity. The CDI-2 has a high level of internal consistency (Cronbach’s alpha = 0 .91), a high test-retest reliability (0.76-0.92) and has correlated positively and significantly with other measures of paediatric depressive symptomology including the Beck Depression Inventory Youth version (BDI-Y) (rs = 0.37) and the Conner’s Comprehensive Behaviours Rating Scales (CRBS) (rs = 0.57) [19].

### Secondary outcome measures

#### The EuroQol group EQ-5D-5 L and EQ-VAS

The EQ-5D is a standardised measure of health-related quality of life (HRQoL) that provides a simple, generic measure of health for clinical and economic appraisal. The EQ-5D-5 L comprises five questions on mobility, self-care, pain, usual activities, and psychological status. For each question there are 5 possible responses, ranging from best to worse. Responses were scored using the published ‘cross walk’ data set [22]. Possible scores for the EQ-5D-5 L range from 1 to −0.594. On this scale 1 is considered equal to full health and 0 is equal to death. The scoring system allows for some health states to be considered ‘worse than death’. As the EQ-5D-5 L is comparatively new the body of literature on its use is developing. However, there is evidence supporting the use in depression of the EQ-5D-3 L (3-level where each question has 3 possible answers). The EQ-5D-3 L was found to be responsive to changes in depression [23], and mean EQ-5D-3 L scores have significantly correlated with a number of clinical outcome measures relating to major depression in adolescents [24].

Following completion of the EQ-5D-5 L participants completed the EQ-VAS. The EQ-VAS is a vertical visual analogue scale that asks respondents to state their current health status as a value between 100 (best imaginable health) and 0 (worst imaginable health).

#### Leisure Time Exercise Questionnaire (LTEQ) [25]

The LTEQ is a self-report questionnaire of leisure time physical activity asking respondents to report the number of times they engage in mild, moderate or strenuous physical activity for at least 15 min over the course of a week [26]. The following categories are then computed: Active; moderately active; and insufficiently active.

The LTEQ is short, easily administered, reliable, has demonstrated concurrent validity with other self-report physical activity tools [27] and has been used with both adult and paediatric participants [28].

### Ethical approval

The study received ethical approval from Nottingham Research Ethics Committee (REC) on 18/07/11. REC reference: 11/EM/0157. Additionally, Research Governance approval was obtained by the Research and Development (R & D) Department of the Trusts (services) from which the participants were identified and recruited.

### Sample size and justification

Exercise is relatively untested in this population, however, based on a Cochrane review of exercise for depressive symptoms with predominantly non-clinical children [13], a medium effect size of 0.50 using Cohen’s d parameters was anticipated [29]. To detect such a difference between two groups at a two tailed 0.05 significance level using 80 % power, 64 participants were required in each arm. After adjusting for 20 % anticipated attrition rates, the required sample size was inflated to 158.

### Data analysis

Data analysis was conducted using the Intention to Treat (ITT) principle. Multilevel regression modelling (MLM) was performed to quantify the treatment effects on the change scores from baseline measures. The following were included as explanatory variables: binary treatment status; actual follow up time; interaction term of treatment by time; baseline CDI-2; and time lag (weeks) between baseline and start of the intervention [30]. Although Multilevel modelling is able to take into account missing data to give sensible results under the missing at random (MAR) assumption [31], sensitivity analysis was also conducted whereby missing values were additionally imputed using an analytical model; the Markov chain Monte Carlo approach [32]. The results between the imputed and observed data were then compared [33]. Multilevel ordinal logistic regression was initially applied to explore the treatment effect for activity level; however, there was no significant level 2 effects shown for this outcome therefore single level models were used to explore the treatment effects on activity level. For all modelling of secondary outcomes, the same set of exploratory variables were included as for the primary outcome. All analyses were conducted using Stata 13.

## Results

**Fig. 2:**
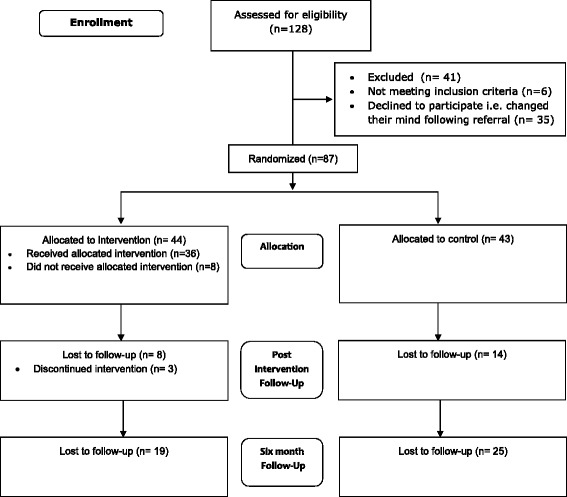
CONSORT flow diagram; a diagram showing the number of participants at each stage of the study process from eligibility assessment to follow-up

The recruitment period ran from May 2012 to September 2013. The flow of patients through the study is given in Fig. 2. In total, 128 participants were assessed for eligibility by the research team; however this is unlikely to reflect the actual number of participants referred to the research team as the clinicians were not able to keep track of this information.

In total, 87 young people were recruited and randomised across the two arms of the trial; intervention (*n =* 44), control (*n =* 43). At post intervention the total loss to follow up was 25 %, with more participants dropping out in the control arm than the intervention. There were no incidents of blinding breach reported by outcome assessors.

A chi-square test of independence was performed to examine the relationship between groups and drop out at post intervention. The relationship between these variables was not statistically significant (*χ*^2^ = 2.59, *p =* 0.11), suggesting that drop out was not affected by allocation to either arm of the trial. At six months, the total loss to follow up was 51 % with a higher dropout rate in the control compared to the intervention arm. However, there were no statistically significant differences in drop out between trial arms at six months (*χ*^2^ = 2.29, *p =* 0.14). Finally, dropout was not predicted by baseline CDI-2 score at post intervention (*p =* 0.74) or six months (*p =* 0.68).

Baseline demographic and clinical characteristics were comparable between groups (see Table 1 and Table 2).

**Table 1:** Participant baseline demographic information

Variables	Intervention (*n =* 44)	Treatment as usual (*n =* 43)
Mean age in years (SD)	15.4 (1.0)	15.4 (0.9)
Gender (%)		
Male	11 (25)	8 (19)
Female	33 (75)	35 (81)
Ethnicity (%)		
White British	42 (95.5)	42 (97.6)
Other (not stated)	2 (4.5)	1 (2.4)
Employment status (%)		
Student	40 (90.9)	40 (93)
Employed	2 (4.5)	0 (0)
Unemployed	2 (4.5)	1 (2.4)
Other (not stated)	0 (0)	2 (4.6)
Living arrangements (%)		
Both natural parents	14 (31.8)	12 (27.9)
Mother and mothers partner	9 (20.5)	9 (20.9)
Father and fathers partner	4 (9.1)	3 (6.9)
Relative or family friend	2 (4.5)	0 (0)
Single parent	14 (31.8)	18 (41.9)
Other (not stated)	1 (2.25)	1 (2.3)

**Table 2:** Participant baseline clinical information

Variables	Intervention (*n =* 44)	Treatment as usual (*n =* 43)
Self-reported treatment type (%)		
Talking therapy in CAMHS^1^	12 (27.3)	10 (23.2)
Counselling alone^+^	18 (40.9)	18 (41.8)
CBT alone^+^	1 (2.3)	0 (0)
Support^+^	1 (2.3)	1 (2.3)
Counselling and medication^+^	1 (2.3)	3 (6.9)
CBT and medication^+^	1 (2.3)	1 (2.3)
Waiting list^+^	6 (13.6)	5 (11.6)
No Treatment	4 (9.1)	5 (11.6)
Psychiatric medication (%)		
Antidepressants	2 (4.5)	5 (11.6)
Hypnotics	2 (4.5)	3 (6.9)
No medication	40 (91)	35 (81.3)
Referring service (%)		
CAMHS Tier 2	30 (68.2)	28 (65.1)
CAMHS Tier 3	10 (22.7)	11 (25.6)
General Practitioner	2 (4.8)	2 (4.7)
School Nurse	2 (4.8)	2 (4.7)
Weekly Physical activity level (%)		
Insufficiently active	26 (61.9)	26 (60.4)
Moderately active	10 (22.7)	6 (14.0)
Active	8 (18.2)	11 (25.6)
Self-harm in previous six weeks (%)		
Yes	11 (25)	15 (34.9)
No	33 (75)	28 (65.1)
^a^Median HrQoL^2^ (IQR)	0.73 (0.23)	0.81 (0.12)
^b^Mean EQ-VAS (SD)	55.1 (20.2)	62.1 (18.2)
Mean CDI-2 Score (SD)	29.1 (9.4)	28.2 (6.8)

^1^Unknown specific modality of treatment

^2^Health related Quality of Life using EQ-5D -5 L

^a^Non-significant difference (*p =* 0.06)

^b^Non-significant difference (*p =* 0.09)

^+^Treatment received through CAMHS

### Primary outcome

Change values on CDI-2 scores at the post intervention period are given in Table 3. Table 3 shows the between group comparison of change from baseline scores (derived from the mulitlevel modelling). For the baseline to post intervention period, the model estimated an additional 2.5 point reduction in CDI-2 score through allocation to the intervention group when compared to the control. However, this difference was not statistically significant (−2.57, 95 % CI −6.82, 1.68, *p =* 0.37).

**Table 3:** multi-level Modelled change from baseline scores and group comparison of change scores

Outcome	Intervention		Treatment As Usual		Multi-level model analysis	*p* value
	Change from baseline (95 % CI)		Change from baseline (95 % CI)		Between group change from baseline (95 % CI)	
CDI - 2						
6 weeks	−5.21 (−8.29, −2.14)		−2.64 (−5.92, 0.63)		−2.57 (−6.82, 1.68)	0.23
6 months	−8.63 (−12.15, −5.12)		−3.82 (−7.53, −0.12)		−4.81 (−9.49, −0.12)	0.03
EQ-5D-5 L						
6 weeks	0.04 (−0.48, 0.57)		−0.33 (−1.07, 0.42)		0.37 (−0.53, 1.28)	0.42
6 months	0.06 (−0.47, 0.59)		−0.30 (−1.02, 0.41)		0.36 (−0.52, 1.26)	0.41
EQ-VAS						
6 weeks	5.59 (−1.08, 12.26)		3.65 (−3.14, 10.44)		1.94 (−7.76, 11.64)	0.69
6 months	6.10 (−1.49, 13.69)		5.40 (−3.29, 14.09)		0.69 (−10.2, 11.60)	0.89
LTEQ	*N*	(%)	*N*	(%)	Probability difference between groups (95 % CI)*	
6 weeks	36		28			
Insufficient active	12	(33)	9	(32)	−0.02 (−0.19, 0.23)	0.87
Moderately active	11	(31)	13	(46)	−0.0 (−0.02, 0.02)	0.91
Active	13	(36)	6	(22)	−0.02 (−0.23, 0.19)	0.86
6 months	25		17			
Insufficient active	18	(78)	15	(88)	−0.23 (−0.53, 0.07)	0.10
Moderately active	0	(0)	1	(6)	0.11 (−0.03, 0.25)	0.09
Active	7	(28)	1	(6)	0.12 (−0.05, 0.29)	0.14

*Ordinal logistic regression with robust standard error adjusting cluster effect due to repeated measures

### Secondary outcomes

The secondary outcomes of the trial were the between group changes scores for the following variables: CDI-2 at six months; HRQOL at post intervention and six months and weekly physical activity level at post intervention and six months.

Modelled differences in secondary outcomes are provided in Table 3. A statistically significant between group change from baseline of CDI-2 scores at six months in favour of the intervention was found (−4.81, 95 % CI −9.49, −0.12, *p =* 0.03).

No statistically significant treatment effects were found for any of the remaining secondary outcomes at post intervention or at six months. However, a statistically significant within group change was observed at post intervention for CDI-2 scores for those in the intervention group.

### Intervention engagement

Of the 44 participants randomized to the intervention arm of the trial, 8 participants (18.1 %) attended no exercise sessions. The reason for non-engagement of these 8 participants was mostly unknown as the majority were non contactable. Only 3 participants gave reasons for non-engagement; exam pressure, high levels of anxiety and feeling too low in mood. The average attendance for all participants allocated to the intervention arm was 66 % (median = 8)

**Fig. 3:**
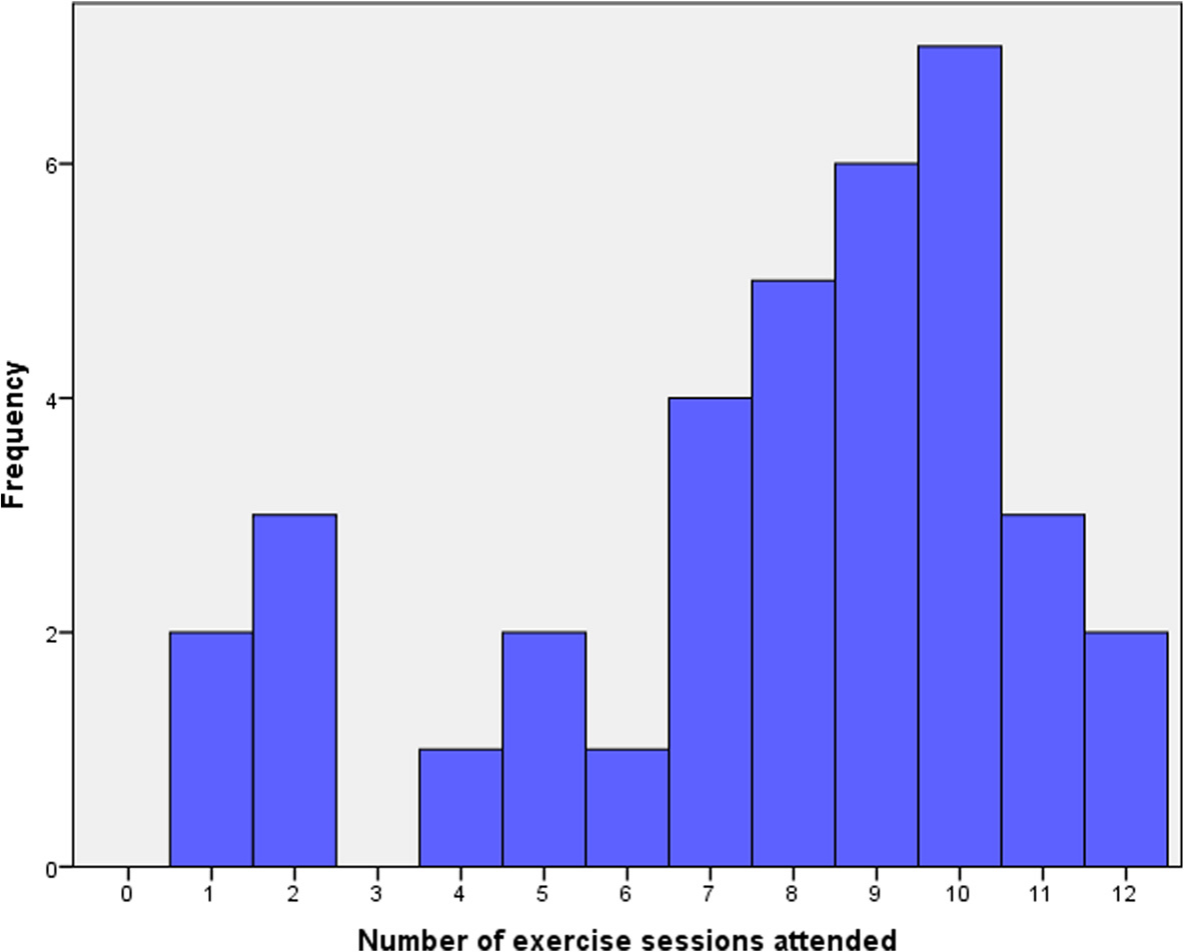
Histogram of session attendance

Of the 36 participants who received the intervention (attended at least 1 session) only 3 participants (8.3 %) dropped out. The average attendance for those who received the intervention was 70 % of scheduled sessions (median = 8.5) (See Fig. 3).

Alongside attendance data, participant engagement with the intervention was measured using heart rate and participant rating of RPE. The mean heart rate and RPE score across all sessions was 103.4 (SD = 15.1) and 10.1 (SD = 1.5) respectively. No statistically significant differences were observed from mid intervention (session 7) to intervention end (session 12) on RPE (*p =* 0.22) or heart rate (*p =* 0.82).

## Discussion

This study aimed to determine the effectiveness of a preferred intensity exercise intervention on the depressive symptoms of adolescents receiving treatment for depression at post-intervention (week 6) and six months follow-up. No effect on depressive symptoms was found at post-intervention, however a statistically significant effect on depressive symptoms was found at six-month follow-up in favour of the intervention.

Previous trials investigating the impact of exercise on depressive symptoms in adolescents have reported mixed results, with some reporting statistically significant treatment effects for depressive symptoms [34–36] and other studies reporting no such treatment effects [37–39]. The current study adds to this body of literature, suggesting no effect of exercise as an additional treatment alongside TAU immediately post intervention.

The likely reason for the non-significant antidepressant effect is that the study is lacking the statistical power required to detect a difference. At the outset, using Cohen parameters [29], a medium effect size was anticipated. As a consequence, our power calculation suggested 128 participants were required in order to have 80 % to detect such a difference. Only 87 participants were recruited, and as such, the difference may have been missed.

A further possible explanation for the lack of a statistically significant treatment effect is that this trial was, in essence, attempting to determine the additional benefit of exercise alongside TAU. No previous studies have investigated the added benefit of exercise for a clinical sample of adolescents receiving mental health treatment. As such, the small effect observed in previous trials may be due to the comparison treatment being substantially less efficacious.

Another potential contributor to the non-significant treatment effect refers to the six-week duration of our intervention; this duration may be viewed as short or medium-term compared to: i) the advice from the National Institute for Care Excellence (NICE) (2005) of a ten-twelve week long structured exercise programme for children and adolescents with depression and ii) a recent RCT of a 12 week exercise intervention reporting antidepressant treatment effects [35]. Moreover, trial participants in this study were required to exercise at their preferred intensity. Given the low self-esteem and self-efficacy levels of people with depression, a number of sessions were used by trial participants to explore and eventually select preferred intensity before building up successful experiences and positive affective responses. Thus, an intervention with a longer duration may have led to higher treatment effects.

Our concomitant qualitative study [40] in which we interviewed 26 participants who completed the intervention arm of the trial revealed that a number of participants’ mood dropped following completion of the intervention. They stated this was due to losing something that improved their mood, provided a distraction, increased their self-efficacy, and improved overall motivation. In contrast, the control arm participants continued TAU and therefore were unlikely to have experienced the loss of a structured intervention, at least not at the time of the post intervention measures. It is proposed that the disappointment and dip in mood experienced as a result of ending their participation in intervention may have temporarily masked any improvement in depressive symptoms.

Considering the statistically significant decrease in depression scores from baseline for those in the intervention group alongside the confidence intervals of the between group difference suggesting that the real difference may be up to seven points in favour of the intervention. The findings suggest that exercise may still hold promise as a treatment for depression in this population.

### Depressive symptoms at six months

A significant treatment effect was observed at six month follow up whereby allocation to the treatment arm was predictive of approximately a five point difference in depressive symptoms, compared to the control arm. The intervention appeared to have a delayed effect on depressive symptoms.

This treatment effect at six months is a particularly novel finding as the majority of previous studies investigating the impact of exercise on depression in adolescents have not included long term follow ups. There have, however, been two notable exceptions. In a large RCT comparing exercise alongside a 50 min educational and cognitive behavioural therapy (CBT) class compared to an equivalent contact, no exercise comparison for high school students, Melnyk et al. [39] found no statistically significant differences between groups on depression scores at six months. However, this may be explained by a potential floor effect owing to the low depressive symptoms observed at baseline.

Conversely, Hughes et al. [35] investigated the impact of a 12 week exercise intervention on the depressive symptoms of adolescents diagnosed with MDD, and included follow ups at 26 and 52 weeks. No statistically significant differences were found on depressive symptoms at either time point. However, the small numbers analysed at 26 weeks (10 vs 9) and at 52 weeks (7 vs 8) potentially explain this lack of treatment effect. In contrast, the statistically significant effect observed in our trial at six months, possibly stems from the larger sample.

There were no significant demographic or clinical differences between participants who were lost to follow up and participants who remained in the study, consequently, it appears that the improvement in depressive symptoms observed at six months may have been attributable to engagement with the exercise intervention. However, no between group differences were observed on the LTEQ at six months, indicating that those in the intervention arm did not continue to exercise above and beyond control participants. As such, it is likely that the mechanism of action may be the additional positive experiences of the intervention reported in the concomitant qualitative study [40] as opposed to increased exercise.

The improvements in depressive symptoms occurring six months post-intervention concurs with the premises of the Transtheoretical Model of Change by Callaghan et al. [41]. In this study it is reported that significant behaviour changes take at least six months to take effect. The time-demanding aspect of the exercise intervention (exploration and selection of the preferred intensity exercise before building up successful experiences and positive affective responses) supports the conclusion Callaghan et al. reported.

When viewed in context, the treatment effect is interpreted as an additional decrease of five points on the CDI-2 over TAU only. The difference in modelled scores from baseline to six-month follow up for the TAU only group was 3.8 points. This suggests that the added effect of an exercise intervention alongside TAU is capable of substantially increasing the depressive reducing effect of TAU alone. Furthermore, when viewing the confidence intervals of the between group comparison, the intervention may lead to a nine point reduction in CDI-2 points. This is likely to be considered clinically meaningful when considering the cut off scale for clinical symptoms of depression is 14 [19].

### Health related quality of life (HRQOL)

No differences were observed between arms on HRQOL as measured by the EQ5D-5 L at either time point. To the authors’ best knowledge, no previous trials in this research area included measures of HRQOL. Consequently there is little data with which to compare the current findings. Nevertheless, there have been studies investigating the impact of exercise on quality of life (QOL) in adults. Importantly, it is acknowledged that HRQOL is not as broad a concept as QOL as its focus is on an individual’s health or disease status opposed to non-health related features of life as well. In a recent systematic review [42] on the impact of exercise on QOL for depressed adults and older adults, exercise was found to improve some QOL domains; primarily the Physical and Psychological domains.

There is some evidence that QOL in depressed adolescents can be improved by current treatment options. For instance, Vitiello et al. [43], found through a large RCT comparing CBT, fluoxetine and a placebo on adolescent depression, a positive effect on QOL for combined CBT and fluoxetine. In light of these findings, we anticipated that a possible explanation for the non-significant difference in HRQOL may be the relatively small sample.

### Intervention engagement

An average attendance of 70 % and drop out of 8 % for participants who attended at least one session, suggest that the intervention was highly acceptable, especially since it was implemented in real life settings (community centres) and no external motives (e.g. vouchers) were provided to participants for attendance. Similarly high adherence rates have been reported by Hughes et al. [35] who conducted an RCT investigating an exercise intervention with depressed adolescents. In this trial, however, participants were given a $25 incentive per session attended, and despite this benefit, adherence typically reduced over time. The high acceptability of our intervention is further supported by the associated qualitative study [40] where the preferred intensity aspect of the intervention is highlighted as one of the key contributing factors regarding intervention adherence.

The importance of preferred activity is further evidenced through analysis of heart rate and perceived exertion data. A mean percentage of maximum heart rate of approximately 50 % and a mean RPE value of approximately 10 suggest that the participants preferred to exercise, on average, at a low intensity. Importantly, the norms through which the RPE is validated were developed for healthy populations [20]. As such, an RPE value of 10 is likely to have a different meaning for this group than the general population. This is considered in light of depression typically being associated with a series of physical symptoms such as aches, tiredness, back pain and gastrointestinal problems [44]. Moreover, the participants in this study were faced with additional physical symptoms due to self-harm and/or other medical conditions (e.g., sciatica or knee joint injuries). Thus, the selected low intensity exercise was unsurprising and through encouraging preferred intensity exercise, the participants did not experience injuries or adverse effects and tended to continue attending the intervention despite the various physical comorbidities seen in the sample.

### Strengths

This study is one of few well-designed trials that have tested the effect of preferred intensity exercise on depressive symptoms in a clinical population of adolescents. The study is a pragmatic RCT, therefore minimum exclusion criteria were employed and the intervention was delivered in a setting reflecting clinical practice. As such, this study has high external validity, as the included participants represented a clinical population, and the intervention was delivered in a ‘real life’ setting. The pragmatic design also allowed for a TAU control condition, thereby reflecting current clinical practice. Consequently, this study is the first to determine the added benefit of an exercise intervention to mental health TAU for depressed adolescents. Our findings, therefore, are of particular interest for researchers and practitioners involved in primary care. Moreover, our study recruited more participants than any previous trial of adolescents using mental health services for the treatment of depression. A six month follow-up period is also rare in studies of this nature with depressed adolescents. The use of preferred intensity exercise applied to populations seeking mental health care and treatment has been pioneered by our group [16] and this is the first such study targeting adolescents living with depression and seeking treatment from mental health services.

### Limitations

Despite being the largest study testing the effect of exercise in depressed adolescents, we were unable to recruit to the required sample size, and this may explain the lack of statistical significance at post-intervention. Moreover, the relatively small sample size increases the risk of a type 1 error which should be considered when interpreting the six month treatment effect. The failure to recruit to the required sample size was due to difficulties in engaging CAMHS and G.P services at the outset of the project, however once the study had been promoted within all the relevant teams, recruitment increased substantially. However, funding was not available to continue data collection beyond the pre-specified end point.

Further limitations may include the relatively short duration of the intervention, the inclusion of exercising young people and the lack of data concerning the amount of exercise being undertaken by participants during the intervention period.

## Conclusions

Preferred intensity exercise in addition to treatment as usual may improve depression outcomes in adolescents living with depression and seeking help from mental health services. However, the improvement in depression may not take effect until at least six months post-intervention. Such findings should be considered with caution considering the relatively small sample size and further research is required in order to confirm these findings.

## Abbreviations

CAMHS: Child and adolescent mental health services
CBT: Cognitive behavioural therapy
CDI-2: Children’s depression inventory 2^nd^ version
CSRI: Client service receipt inventory
EQ-5D-5 L: Euroqol 5 dimensions 5 levels
EQ-VAS: Euroqol visual analogue scale
G.P: General practitioners
HRQOL: Health related quality of life
ITT: Intention to treat
LTEQ: Leisure time exercise questionnaire
MAR: Missing at random
MDD: Major depressive disorder
MLM: Multilevel regression modelling
NICE: National institute for health and care excellence
RCT: Randomised controlled trial
R&D: Research and development
REC: Research ethics committee
RPE: Rating of perceived exertion
TAU: Treatment as usual
